# Proposal for an Alliance Between Healthcare and Legal Area Professionals for Shared Public Health and Preventive Strategies in Italy and Europe

**DOI:** 10.3389/fpubh.2020.00324

**Published:** 2020-07-23

**Authors:** Claudio Costantino, Walter Mazzucco, Vincenzo Restivo, Ida Iolanda Mura, Gaetano Maria Fara, Giuseppe Giammanco, Sabrina Vecchio Verderame, Giuseppe Alessio Messano, Carmelo Massimo Maida, Alessandra Casuccio, Francesco Vitale

**Affiliations:** ^1^Department of Health Promotion Sciences, Maternal and Infant Care, Internal Medicine and Medical Specialties, University of Palermo, Palermo, Italy; ^2^Department of Medical, Surgical and Experimental Sciences, University of Sassari, Sassari, Italy; ^3^Department of Public Health and Infectious Disease, University of Rome “La Sapienza”, Rome, Italy; ^4^University of Catania, Catania, Italy; ^5^University of Palermo, Palermo, Italy

**Keywords:** multidisciplinary approach, healthcare disputes, mandatory vaccination, cancer screening, environmental health surveillance, hospital management

## Abstract

Following the implementation of the new Italian legislation on responsibility of healthcare workers, a multi-professional framework, involving representatives of the Italian public health professionals and legal professionals expert in the field, drafted a proposal of the actionable recommendations to be implemented in the management of civil and penal disputes arising from the practice of public health interventions. In order to prevent legal disputes concerning some public health fields such as vaccinations, cancer screening, environmental health surveillance, and hospital management, it should be primary taken into account to update guidelines in supporting decision-making processes, in accordance with the “best scientific evidence available.” Furthermore, a multidisciplinary alliance between public health and legal area professionals should be encouraged and should be promoted both at national and European level.

## Introduction

In 2017 the Italian Parliament approved the Law n.24 “Provisions on care and patients safety, as well as on professional responsibility of healthcare workers” (1) with the aim to reduce avoidable harm in healthcare (2). The great regulatory uncertainty on patient safety and health professionals' responsibilities documented in Italy before the introduction of this reform was one of the reasons for the increase of disputes in the medical sector and was considered responsible for the economic burden of unsafe care (3). Together with the billionaire compensation attributable to adverse events (4) and to the widespread defensive medical practices (5), they both represented the main drivers of the new law. For the previous reasons, the main objective of this new regulation was to harmonize the relationship between doctors and patients by monitoring the medical malpractice cases and disputes, drafting preventive measures, as well as reducing the level of litigation (3). However, despite the protection of health professionals' liability when complying with guidelines and safe practices make it possible to take a legal action against healthcare professionals only in cases of malice or gross negligence (2), some concerns still remain in the real-world both from a civil and a criminal point of view.

Against the backdrop of the latest jurisprudential developments resulting from the approval of the mentioned law, a course entitled “The civil and penal liability in the exercise of Public Health and related health-care professions,” the 52nd organized by the “Giuseppe D'Alessandro” International School of Epidemiology and Preventive Medicine at the “Ettore Majorana” Foundation and Scientific Culture Center (6), was held in Erice (Trapani) from the 2nd to the 6th of May, 2018. Courses at “Ettore Majorana” international center focus on case studies dealing with current and innovative topics. At the end of each course a declaration is provided with the aim to spread the results of an interactive process between a panel of high-skilled experts, discussants, and attendees. In particular, all of the 52nd Erice course participants, representatives of the Italian public health professionals, together with legal professionals expert in the field, have been called to widely debate in order to draft a proposal of actionable recommendations to be taken in the management of civil and penal disputes arising from the practice of public health interventions (1). Whereas, both the public health and preventive medicine sectors have several areas of influence and intervention (such as vaccination, cancer screening, environmental and community health, hospital management, public health, and prevention measures, etc.), the development of a multidisciplinary framework, including the juridical expertise, was considered of strategic importance to share different experiences and point of views.

## Policy Options and Implications

### Vaccinations

In Italy, the Law n.119 of June 2017—“Urgent measures regarding vaccination”—introduced mandatory vaccination for school enrollment toward 10 infectious diseases (diphtheria, tetanus, pertussis, hepatitis B, haemophilus influenzae type B, measles, mumps, rubella, chickenpox) (7). This legal provision has been established to guarantee “herd immunity” and to contrast the substantial drop of vaccination coverage observed in Italy in the last decade, that contributed to a large outbreak of measles in 2017/2018 (7–9).

This law became an essential tool for public health professionals, pediatricians, and general practitioners to raise vaccination coverage able to reach “herd immunity” levels and reduced the circulation of vaccine preventable diseases (VPDs), whatever in Italy, despite the international recommendations for immunization of health-care workers (HCW's) and the National Vaccination Plan (NVP) 2017–2019, vaccination coverage of HCW's for VPDs are lower than recommended (10–13). Moreover, the absence of mandatory vaccination measures for HCW's in Italy could put a limit on the implementation of the Gelli-Bianco Law by not guaranteeing health-care safety, risk management nor prevention (14–16).

The current lack of mandatory vaccination laws for HCW's, also in case of VPDs transmission to patients that eventually results in death or serious injury, make any responsibility of the HCW's not be punishable.

Otherwise, recalling the aforementioned recommendation of the Vaccination Plan, the contractual responsibility of the healthcare facility in which the HCW operated should be evaluated and considered in the preventive regulation for health service performances.

### Cancer Screening

A further preventive topic which could lead to legal disputes is represented by oncological screening. In the last 10 years, the effectiveness of screening programmes led international and national public health institutions to consider it as a fundamental strategy of secondary prevention also in reducing health-related costs (17, 18).

The Italian legislation considered screening programmes as essential levels of assistance for the general population, namely preventive interventions to be granted actively and free of charge, offered to all citizens residing in Italy (19, 20).

The legal dispute could derive from the occurrence of any organizational and structural deficits such as: extension and delivery of invitation letters, provision and supply of diagnostic services and activities, or possible presence of false positives and false negatives, having a negative impact on the National Health Service (NHS) (21, 22).

### Environmental Health Surveillance

Other responsibility profiles could arise in other sectors of public health, such as environmental protection and surveillance, given that environmental problems have important repercussions on the population's health. Also, in this area, some serious legislative gaps on the regulation of well-defined qualitative and quantitative limits for emerging pollutants have arisen in the European and Italian context.

More recently, environmental emergencies due to perfluoroalkylated substances in the North East of the Italy (Veneto region) (23) and, in the South Italy, to polychlorinated biphenyls (PCBs) dioxins-specific (Taranto, Apulia region) (24) and to pollutants emitted from waste management on fire in Sicily island (25), reaffirmed the need to plan preventive controls and strategies to reduce any types of pollutants in the environmental media and to establish and legislate, based on the most recent scientific evidence, any limits to exposure, if not yet defined.

### Hospital and Risk Management

Finally, the legal responsibility profiles in the hospital and risk management were debated during the course. Specifically, the main criticism which emerged was the lack of a national legislation for assignment of health directions (in particular, sanitary directions) to health professionals with the medical specialization in hygiene and preventive medicine (26). Instead, medical and non-medical professionals outside the public health area were frequently assigned to those roles, which could potentially expose a lower guarantee of appropriateness and effectiveness of the services provided in the NHS (26).

### Actionable Recommendations: The Erice Declaration

The actions to be taken in the management of civil and penal disputes arising from the practice of public health interventions, as proposed at the end of the multidisciplinary and interactive process, are listed as follows.

1. Laws on mandatory vaccination demonstrated a substantial increase of vaccination adherence when implemented (27). At the same time, also mass media should be addressed carefully with regard to vaccination issues for supporting this public health strategy (28, 29). In the future, public health HCW's should represent, in alliance with pediatricians and general practitioners, a guide for other health professions and for mass and social media in the promotion of a vaccination culture among the general population.
  Moreover, synergies with legal players that had a role in questionable judicial decision during past years should be strengthened (30). These claims, uncontrollably magnified by media, contributed to the decrease of vaccine confidence and vaccination adherence.
2. The mandatory evaluation of the HCW's immunization status operating within the National Health Service did not increase vaccination coverages for VPD's among them. Expecting future national and regional regulations of mandatory vaccination for HCW's, a multilevel approach with the adoption of the following strategies could be evaluated:
  – An informative form for patients specifying strategies adopted in single hospital and territorial units to prevent VPD's;
  – Formative/informative programmes for HCW's on VPD's transmission modality and preventive strategies before the organization of vaccination campaigns (12, 31);
  – Active offer of recommended vaccination in the NVP for patients entering in outpatient setting or patients in resignation from hospital units, registering the vaccine information update in the discharge record;
  – Evaluation of incentive/disincentive actions by hospital and primary healthcare management for the promotion of vaccination among HCW's (32);
  – Promotion of a surveillance system for procedures and strategies adopted in the prevention of healthcare-associated infections due to VPD's (15, 16).
3. Cancer screening programmes require a considerable organizational effort of prevention departments (17, 18). In order to guarantee the highest level of invitation letters to the extension and adherence of the target population, a major involvement of HCW's operating in territorial medicine (e.g., GP's), especially for the recovery of subjects difficult to reach by the current invitation systems, should be considered (20, 33). Not only should the minimal organizational, structural, and technological requirements be guaranteed, but also the highest levels of sensibility and specificity of the screening programmes throughout the constant update of diagnostic methodologies based on the health technology assessment (HTA) procedures (34).
4. Moving forward, environmental health should take the environmental impact of the various productive activities and the effects on the health of the community into consideration. To reach this objective, all European Nations should standardize and regulate the application of tools such as the evaluation of health impact from prevention departments of the local health units to plan controls and prevent environmental emergencies, which would otherwise be more complex to manage.
5. In order to improve the performances of hospital and primary health care departments, it would be desirable to allocate these functions to health professionals who specialize in hygiene, preventive medicine, and to train students of public health in Italian post-graduate schools (24).

## Future Perspectives and Conclusions

Italy is one of the few countries in Europe having already enacted a comprehensive law on patient safety, preserving at the same time patients' rights to safe care and fair compensation in case of harm, and transparency (35). The Italian reform law on responsibility of healthcare workers (L. 24/2017) represents a bold step toward the goal of reducing avoidable harm in healthcare and recognizes the central role of guidelines in support of decision-making in the health sector (2). To this end, it stated that guidelines need to be implemented in collaboration with accredited scientific societies through the Italian National Guidelines System on behalf of National Institute of Health (36). Anyway, since the first steps of the reform implementation arose the need to fill the existing cultural gaps making a bridge between representatives of the Italian public health professionals legal professionals expert in the field.

In 2005, a comparative study of the legal and factual situation on Medical Liability in six Member states of the European Council was conducted (37). Moreover, in France, since the introduction of the Patients' Rights Law, in 2002, a regulation on Medical Liability was introduced (38). However, in other European Countries and in Italy, to date and to the best of our knowledge, there is a lack of guidelines and recommendations for civil and penal disputes management arising from the practice of public health interventions.

Within this framework, the faculty (speakers, rapporteurs, and discussants) of the 52nd course of the “Giuseppe D'Alessandro” International School of Epidemiology and Preventive Medicine unanimously, in agreement with all of the attendees, recognized the opportunity to promote a permanent multi-professional alliance to develop the “best scientific evidence-based guidelines” following a multidisciplinary approach in the resolution of legal disputes concerning public health. The Italian Society of Hygiene, Preventive Medicine and Public Health was indicated as the proper interactive platform where to convoy the different point of views and expertise, particularly through the working groups dedicated to the topics treated during the course (vaccination, screening, environmental health, etc.), so to update the existing national guidelines and to propose new ones oriented on the “best scientific evidence available.” These “revised” guidelines could contribute to provide additional guidance for public health professionals and judgment tools in the public health field for legal professionals. Last, but not least, this multi-professional alliance should be exported at an international level and broadly promoted through the European Public Health Association network.

A permanent alliance between healthcare and legal area professionals for shared Public Health and Preventive strategies in Italy and Europe is proposed.

## Author Contributions

All individuals listed as authors have contributed to designing, performing or reporting the study and every specific contribution is indicated as follows. CC, WM, GG, GF, and FV: conception and design of the study. GF, FV, IM, and AC: supervision. CC and WM: manuscript writing and drafting. CC, WM, VR, IM, GF, GG, SV, GM, CM, AC, and FV: revision of the manuscript. CC, WM, VR, IM, GF, GG, SV, GM, CM, AC, and FV: approval of the final version of the manuscript.

## Conflict of Interest

The authors declare that the research was conducted in the absence of any commercial or financial relationships that could be construed as a potential conflict of interest.
